# Constrained Forest Methane Sink by Thermal Adaptation of Soil Methanotrophs

**DOI:** 10.1002/advs.77426

**Published:** 2026-08-25

**Authors:** Baizhi Jiang, Xi Zhang, Zhenghu Zhou, Nianpeng He, Xuhui Zhou, Hongyang Chen

**Affiliations:** ^1^ Institute of Carbon Neutrality School of Ecology Key Laboratory of Sustainable Forest Ecosystem Management‐Ministry of Education Northeast Forestry University Harbin China; ^2^ Key Laboratory of Boreal Forest Ecosystem Conservation and Restoration National Forestry and Grassland Administration Northeast Forestry University Harbin China

**Keywords:** climate warming, microbial methane oxidation, thermal adaptation

## Abstract

Climate warming can stimulate soil methanotroph‐mediated CH_4_ oxidation, thereby mitigating the increase in atmospheric CH_4_ concentrations in response to anthropogenic activities. However, the positive response of microbial activity to warming often weakens over time owing to thermal adaptation. Whether soil methanotrophs also show such adaptation in response to warming remains unknown, adding considerable uncertainty to predictions of soil CH_4_ sink in a warming world. Here, we collected soils from 67 forest sites spanning a broad thermal gradient to investigate the response of microbial CH_4_ oxidation to long‐term temperature change. We find that microbial CH_4_ oxidation rates decrease with rising mean annual temperature, indicating that soil methanotrophs thermally adapt to long‐term temperature change. Our results further show that the thermal adaptation of microbial CH_4_ oxidation decreases following long‐term exposure to experimentally elevated CH_4_ concentrations. Our findings suggest that the enhancement of soil microbial CH_4_ oxidation rates resulting from warming may be smaller than previously predicted and that considering thermal adaptation of microbial CH_4_ oxidation under diverse CH_4_ concentrations is crucial for accurate predictions of future soil CH_4_ sinks.

## Graphical abstract

Climate warming can stimulate methanotroph‐mediated CH_4_ oxidation in forest soils, thereby mitigating the increase in atmospheric CH_4_ concentrations. By integrating evidence from a macro‐climatic thermal gradient and a 70‐day incubation experiment, this study demonstrates that microbial methanotrophs can adapt to long‐term temperature changes at the community level, constraining the expected increase in forest soil CH_4_ sinks.

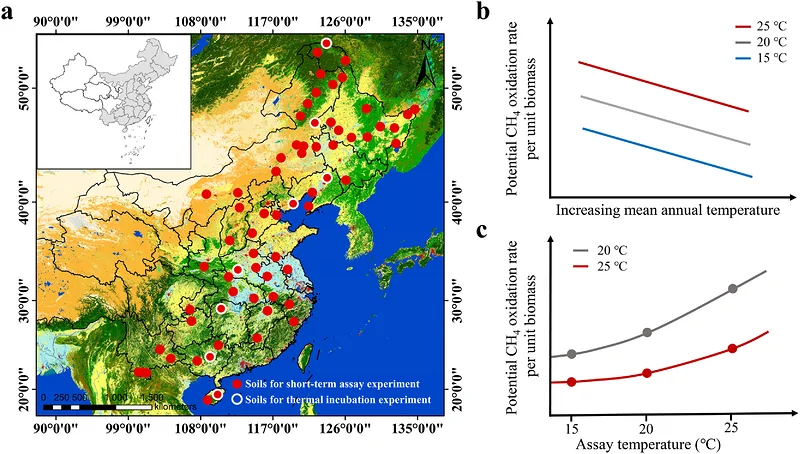

## 1 Introduction

Methane (CH_4_) is a highly potent greenhouse gas that has increased rapidly from pre‐industrial era levels of approximately 0.7 ppm to current levels exceeding 1.8 ppm [1, 2], contributing greatly to global warming [3]. Microbial methanotrophs can oxidize CH_4_, thereby playing a crucial role in the terrestrial soil CH_4_ sink [4, 5]. Observations from short‐term experiments have demonstrated that soil microbial CH_4_ oxidation rates increase with temperature [6, 7, 8], which could enhance the terrestrial soil CH_4_ sink, potentially triggering a negative feedback that may slow the rate of global warming [9]. However, the strength of this negative feedback between the CH_4_ sink and climate warming remains uncertain [8, 10], primarily because the response of microbial metabolism to long‐term temperature change can differ from its instantaneous response [11, 12, 13].

While long‐term warming studies show initial increases in soil microbial metabolic rates, these often quickly return to pre‐warming levels or even decline [14, 15, 16]. This phenomenon, referred to as thermal adaptation, could result from physiological responses of individuals, genetic variation within species and/or shifts in the community composition [17, 18, 19, 20, 21]. Given the increasing evidence that indicates thermal adaptation should be a common property of all organisms [18, 19, 22], it is likely that long‐term temperature change causes adaptive change in microbial CH_4_ oxidation rates. Yet, clear evidence for such a phenomenon is lacking, which adds considerable uncertainty for how warming influences the magnitude of the CH_4_ sink in a warming world.

**FIGURE 1:**
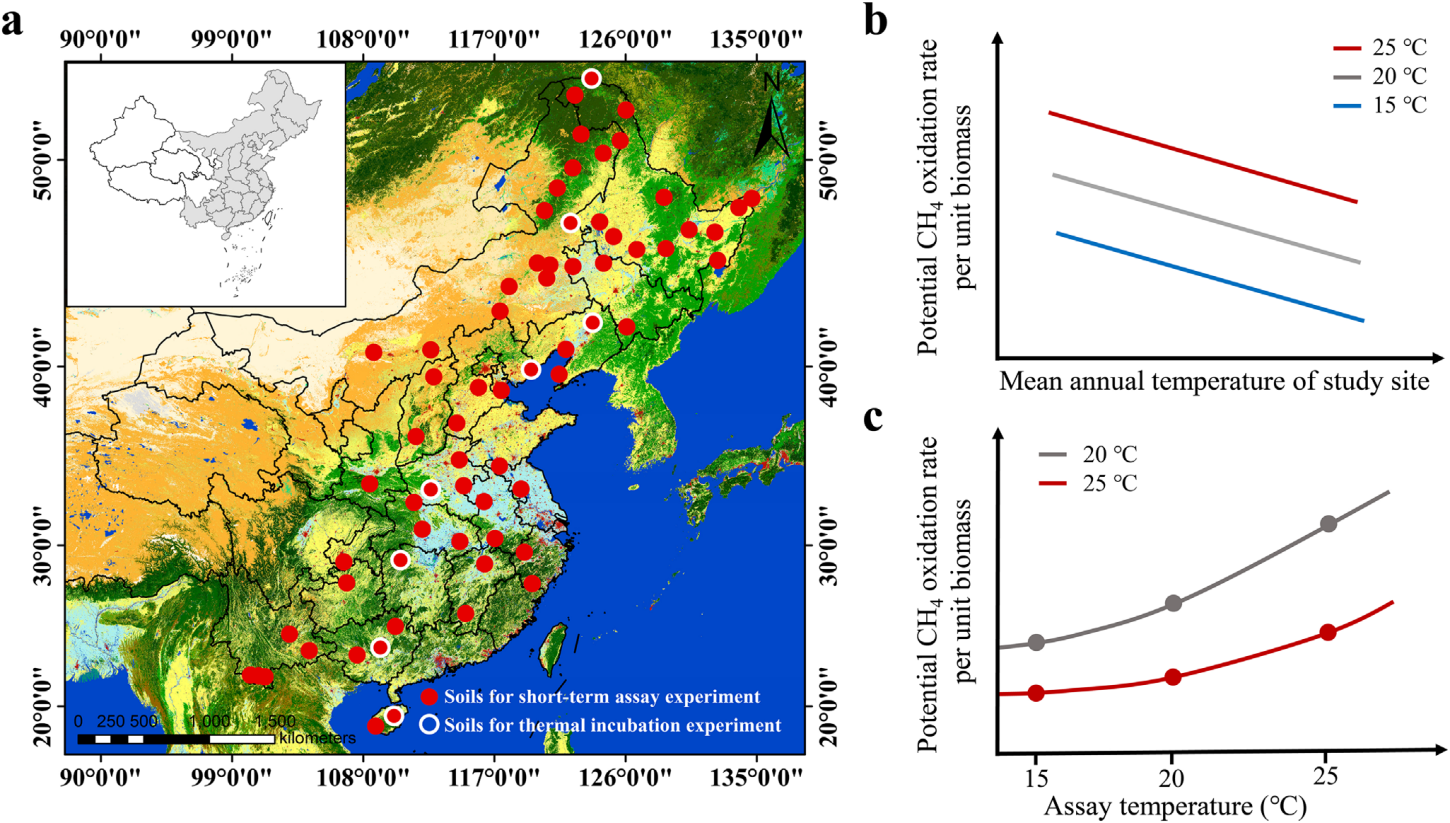
Locations of the studied forest sites and conceptual paradigm of thermal adaptation in microbial CH_4_ oxidation to long‐term temperature change. (a) Geographic distribution of 67 forest sites along a 4000 km north‐south transect in eastern China. (b) We employed the differences in thermal regimes across 67 forest sites to evaluate the thermal response of soil microbial CH_4_ oxidation to long‐term temperature change. If microbial CH_4_ oxidation is thermally adaptable, the rates of soil microbial CH_4_ oxidation per unit biomass decrease with increasing mean annual temperature. (c) Given that methanotroph activity is constrained by atmospheric CH_4_ concentrations, we conducted a 70‐day thermal incubation experiment using soils from eight sites with different thermal regimes to test the thermal adaptation of soil microbial CH_4_ oxidation under low versus high CH_4_ concentrations. This incubation length has been shown to be sufficient for soil microorganisms to adapt to the temperature change [10, 27, 28, 29]. If thermal adaptation occurs, the rates of soil microbial CH_4_ oxidation per unit biomass decline with prolonged exposure to warmer conditions (i.e., microbial CH_4_ oxidation rates per unit biomass in soils incubated at 25°C were consistently and significantly lower than those in soils incubated at 20°C).

Spatial climate–biotic relationships offer valuable insights into ecological responses to climate change [23, 24, 25], as they can serve as proxies for predicting long‐term dynamics, exploiting spatial variability to infer temporal responses of microbial CH_4_ oxidation to warming. Here, we collected soil samples from 67 forest sites along a ∼4000 km north‐south transect in eastern China (Figure 1a). Mean annual temperatures from these sites ranged from −4.57°C to 24.19°C (Table S1), making this transect an ideal case study to test the thermal response of microbial CH_4_ oxidation to long‐term temperature change. Following the approach of previous microbial thermal adaptation studies [23, 24], we eliminated the substrate and moisture limitation on methanotroph activity and measured the microbial CH_4_ oxidation rates from 67 sites at three temperatures (i.e., 15, 20, and 25°C) to investigate whether microbial CH_4_ oxidation adapts to long‐term temperature change (see Experimental Section). Considering that methanotroph activity is limited by low atmospheric CH_4_ concentrations [8, 26], we further examined whether long‐term exposure to low versus high CH_4_ concentrations influences the thermal adaptation of microbial CH_4_ oxidation. We incubated soils from 8 different thermal regime sites at atmospheric CH_4_ concentration (2 ppm) and elevated CH_4_ concentration (500 ppm) under two temperatures (20 and 25°C) for 70 days (see Experimental Section)—a duration proven sufficient to induce metabolic temperature adaptation in microbes [27, 28, 29]. Under elevated CH_4_ concentrations, forest soil methanotrophs are less constrained by substrate availability, and the substrate‐sufficient conditions generally could attenuate the adaptive adjustments of soil microbes to long‐term temperature change [30]. The specific hypotheses we tested in this study are: (1) soil microbial CH_4_ oxidation can adapt to long‐term temperature change (Figure 1b,c). (2) the magnitude of this adaptation is reduced under sustained exposure to elevated CH_4_ concentrations.

## 2 Results and Discussion

**FIGURE 2:**
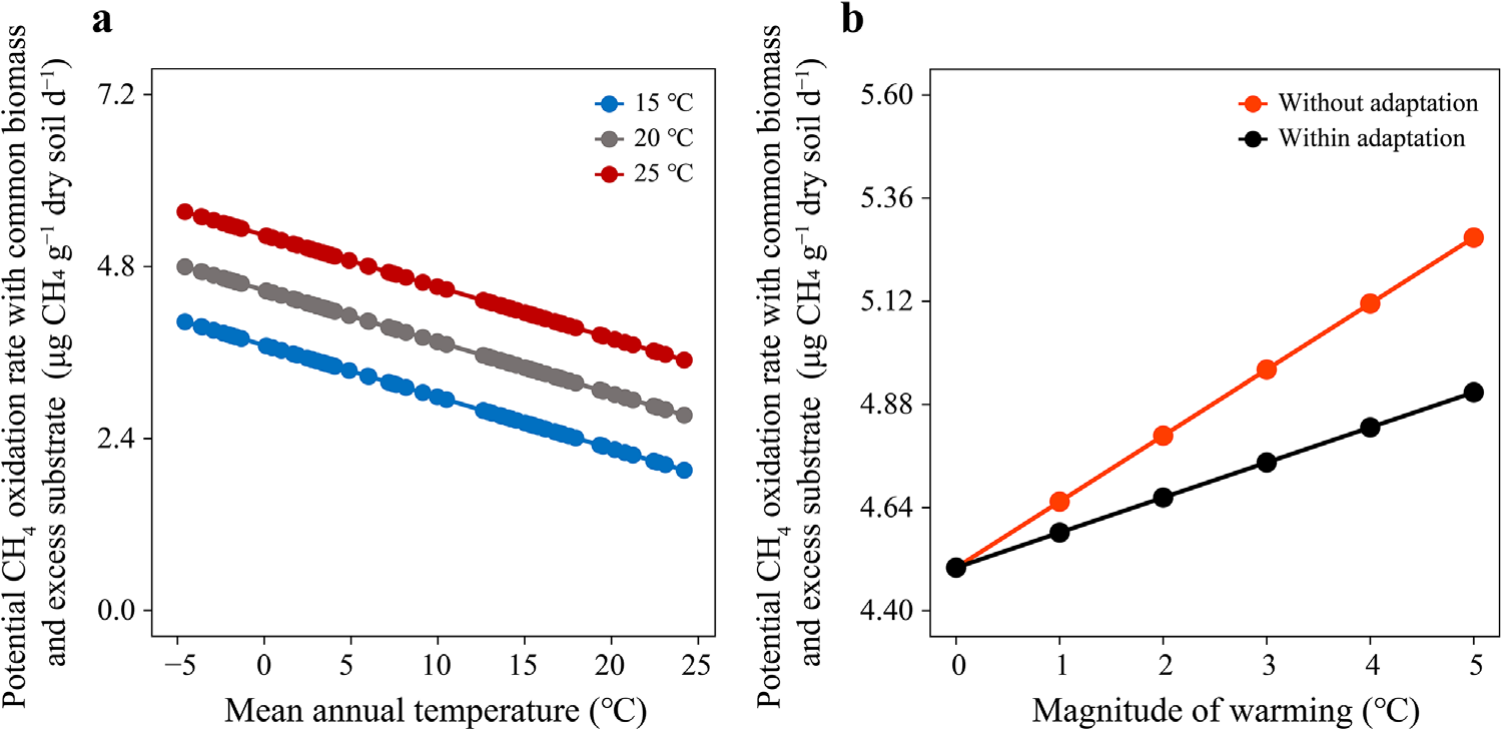
Estimated potential microbial CH_4_ oxidation rates. Model‐predicted potential microbial CH_4_ oxidation rates at a standardized methanotroph biomass and excess substrate. (a) Lines show partial regression fits of mean annual temperature from the mixed‐effects model, with other variables held at their mean values. Points represent model‐based estimates across the observed mean annual temperature range, shown separately for each assay temperature (15, 20, and 25°C). (b) Potential microbial CH_4_ oxidation rates were estimated with temperature increases under scenarios with and without microbial thermal adaptation. In the microbial thermal adaptation scenario, estimates incorporate the negative slope of mean annual temperature from the mixed‐effects model, reflecting long‐term microbial adjustment. Conversely, in the scenario without microbial thermal adaptation, this slope was set to zero (see Experimental Section).

Our analyses revealed that mean annual temperature is a strong predictor of soil microbial CH_4_ oxidation rates across a wide geographical scale, as evidenced by the linear mixed‐effects model (Table S2). In general, thermal adaptation of the microbial community is defined as a decrease in microbial metabolic rates per unit biomass in response to a prolonged increase in temperature [14, 19]. We estimated soil microbial CH_4_ oxidation rates at a controlled microbial biomass in our regression model, which is conceptually analogous to evaluating microbial CH_4_ oxidation rates per unit biomass (see Experimental Section). Our results showed that the potential soil microbial CH_4_ oxidation rates decreased with increasing mean annual temperature across assay temperatures (Figure 2a). This negative relationship suggests that methanotrophs at the community level adapt to a warmer climate, broadly consistent with theoretical expectations (Figure 1b).

To reveal the potential impact of temperature increases on microbial CH_4_ oxidation rates, we used the slope coefficient for the mean annual temperature term from the linear mixed‐effects model to represent the weakening of microbial CH_4_ oxidation rates caused by the methanotroph thermal adaptation. In comparison, this coefficient was set to zero to simulate the scenario without thermal adaptation. In both scenarios, we found that the potential microbial CH_4_ oxidation rates increased with rising temperatures, but were considerably weaker when microbes were allowed to adapt (Figure 2b). Moreover, the dampening effect of thermal adaptation on potential microbial CH_4_ oxidation rates increases with the magnitude of warming (Figure 2b). Given that the magnitude of climate warming is globally variable [31, 32], it will be necessary to consider the magnitude of regional warming when assessing changes in the global soil CH_4_ sink in a warming world.

**FIGURE 3:**
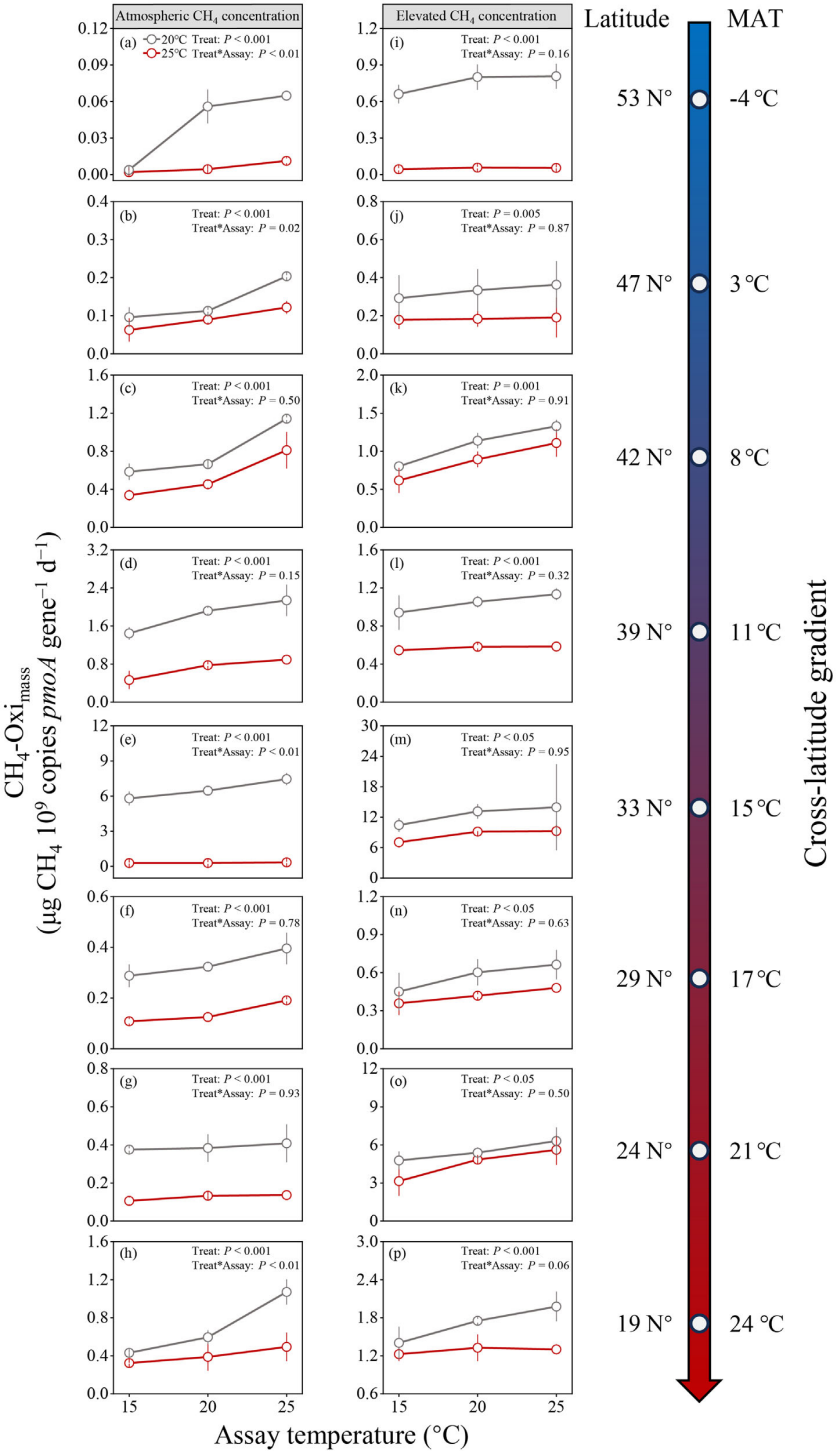
Responses of soil microbial CH_4_ oxidation rates per unit biomass (CH_4_‐Oxi_mass_) to long‐term temperature change across a wide latitudinal gradient. (a–h) Atmospheric CH_4_ concentration treatment (2 ppm). (i–p) Elevated CH_4_ concentration treatment (500 ppm). A two‐way ANOVA was used to assess the effects of incubation and assay temperature on each site's CH_4_‐Oxi_mass_. MAT, mean annual temperature. Values are means with the standard deviation, n = 4.

**FIGURE 4:**
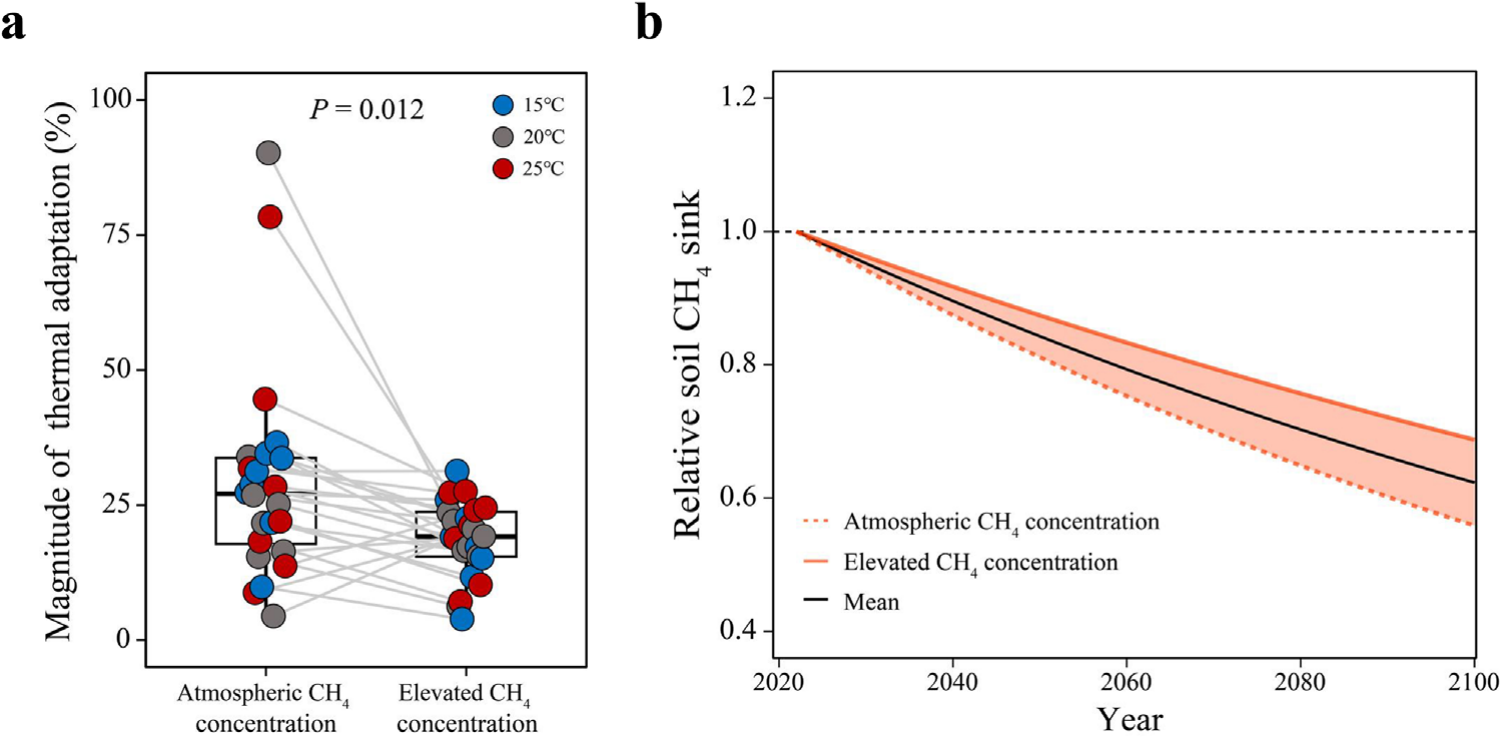
Magnitude of thermal adaptation of microbial CH_4_ oxidation under different CH_4_ concentrations. (a) The magnitude of thermal adaptation of microbial CH_4_ oxidation was compared between the atmospheric CH_4_ and elevated CH_4_ concentration treatments. Different colors represent the magnitude of thermal adaptation of microbial CH_4_ oxidation calculated for three assay temperatures. Statistical significance was tested using a paired two‐tailed *t*‐test. Horizontal lines within the box denote the median, with the box limits representing the upper and lower quartiles. The whiskers extend to 1.5 times the interquartile range. (b) Effects of methanotroph thermal adaptation on the soil CH_4_ sink across China's forests in response to a gradual increase of 2.0°C from 2023 to 2100. Two scenarios were examined: atmospheric CH_4_ and elevated CH_4_ concentrations. The black solid line represents the average of the two scenarios, while the shaded region indicates the range between them. The black dashed line represents the current soil CH_4_ sink.

Following a 70‐day thermal incubation experiment, we found that microbial CH_4_ oxidation rates per unit biomass in soils incubated at 25°C were consistently and significantly lower than those in soils incubated at 20°C, irrespective of long‐term exposure to either atmospheric or elevated CH_4_ concentrations (Figure 3). Our findings illustrate that the thermal adaptation of microbial CH_4_ oxidation persists independently of the available CH_4_ concentration. We also calculated the magnitude of thermal adaptation of microbial CH_4_ oxidation, reflecting the extent to which the expected increases in microbial metabolic rates due to warming are mitigated by microbial adjustments [33, 34]. The results revealed no significant relationship between the magnitude of thermal adaptation of microbial CH_4_ oxidation and mean annual temperature (*p* > 0.05; Figure S1), suggesting that the capacity for thermal adaptation in methanotrophic communities is largely independent of local climatic temperatures. Further analysis showed that the magnitude of thermal adaptation of microbial CH_4_ oxidation was considerably lower when CH_4_ concentrations were elevated (Figure 4a). Changes in community structure are often considered as the key mechanism in regulating thermal adaptation of soil microbial metabolic rates [19, 20]. Aerobic methanotrophs in forest soils are generally classified into type I and type II, with type I characterized by rapid growth rates and a preference for high‐nutrient environments, whereas type II exhibits a much higher affinity for CH_4_ [35]. Previous studies have shown that elevated CH_4_ concentrations can induce a shift in methanotrophic community composition from type II toward type I [36]. Taken together with our finding of reduced thermal adaptation under high CH_4_ concentrations, this evidence suggests that type I methanotrophs may be less capable of adapting to changing climatic conditions than type II methanotrophs.

The factors associated with the magnitude of thermal adaptation of microbial CH_4_ oxidation were examined using random forest analyzes, we found the strongest influence coming from methanotroph richness under atmospheric CH_4_ concentration (Figure S2a). Here, the magnitude of thermal adaptation was negatively correlated with methanotroph richness (Figure S2b). One possible explanation for this relationship between methanotroph richness and thermal adaptation magnitude may be derived from the diversity‐stability hypothesis, which posits that higher species richness enhances the stability of community functions in the face of environmental disturbances [37, 38, 39]. On the other hand, when CH_4_ concentration was elevated, the strongest influence on the magnitude of thermal adaptation was soil total nitrogen content (Figure S2c), with an increasing magnitude of thermal adaptation with soil total nitrogen content (Figure S2d). This relationship could be explained as a result of increasing nitrogen levels leading to higher levels of competition between CH_4_ and NH_4_^+^ for the same methane mono‐oxygenase (MMO) enzyme [40, 41], which is essential for the CH_4_ oxidation process [35, 42].

Using these experimental data, we predict that a 2°C increase in temperature by 2100 may weaken the soil CH_4_ sink by 31% (under elevated CH_4_ concentration) to 44% (under atmospheric CH_4_ concentration) (Figure 4b). Although this range of scenarios was not intended to represent predicted futures, it provides a visualization of how the thermal adaptation of microbial CH_4_ oxidation, under fluctuating CH_4_ availability, can influence the soil CH_4_ sink in a warming environment. Models that accurately capture the long‐term response of microbial CH_4_ oxidation to changes in global temperature can offer a more robust projection of the terrestrial CH_4_ sink‐climate feedback.

Unlike most microbial groups that decompose organic carbon, methanotrophs are unique in their ability to use CH_4_ as their sole carbon and energy source [43, 44]. By providing an adequate supply of CH_4_, we effectively eliminated the substrate limitations on methanotroph activity and provided solid evidence that microbial groups can reduce their metabolic rates to adapt to long‐term temperature change. Indeed, soil ecosystem functions are driven by various microbial functional groups [45, 46, 47]. A recent study found that slow‐growing microorganisms (i.e., *K*‐strategy microbial group) are better adapted to warming than fast‐growing microorganisms (i.e., *r*‐strategy microbial group) [48], indicating that the thermal adaptation of microbial metabolism is regulated by diverse microbial functional groups. Together with our findings, this highlights that focusing on microbial functional groups rather than taxonomic structure can provide a better framework for understanding microbial‐driven carbon‐climate feedback. Building on this functional‐group perspective, it is equally important to consider how environmental factors other than temperature, such as soil moisture, may interact with warming to shape the thermal adaptation of these groups. Although this study demonstrated that forest soil methanotrophs can thermally adapt to long‐term warming under optimal moisture, future climate change may bring concurrent precipitation shifts that alter soil moisture. Elucidating how these moisture changes affect the thermal adaptation of microbial CH_4_ oxidation therefore remains a key priority for understanding how coupled hydrothermal changes regulate the thermal adaptation of soil microbial functional groups in a changing climate.

Overall, our results demonstrate that soil methanotrophs do not simply accelerate their metabolic rate with rising temperatures, but actively adjust their activity in ways that dampen the CH_4_ sink. The strength of this dampening effect depends not only on the thermal regime, but also on the resource context, with methanotroph diversity and soil nitrogen availability emerging as critical regulators. These findings suggest that the capacity of forests to buffer atmospheric CH_4_ increases will be smaller than often assumed, and that the microbial lever in the climate system may tighten rather than loosen under continued warming. Consequently, explicitly integrating microbial thermal adaptation into biogeochemical models, alongside key ecological drivers such as functional group composition and nutrient interactions, will be essential to anticipate the pace of future climate change. More broadly, our study points to the need to treat microbial functional groups not as passive responders but as active agents shaping biosphere–atmosphere feedbacks, opening new directions for predicting, managing, and perhaps even harnessing microbial processes in a warming world.

## 3 Experimental Section

### 3.1 Study Area and Soil Sampling

We collected 268 topsoil (0–10 cm) samples from 67 forest sites along a ∼4000 km south‐north transect in eastern China between June and August 2023. The sites spanned a wide range of latitudes (18.7°−53.4°N) and longitudes (100.3°−134.7°E). The mean annual temperature ranges from −4.57 to 24.19°C and the mean annual precipitation varies from 217 to 1858 mm. Detailed information about each site is provided in Table S1.

At each forest sampling site, we randomly established four 10 × 10 m sampling plots separated by more than 100 m from each other. In each plot, we took six soil core samples (0‐10 cm) following a zigzag path and then mixed them to form a composite sample. The samples were sieved through a 2 mm mesh and divided into three subsamples. One subsample was stored at 4°C until the experiment began, another was stored at ‐80°C for microbial DNA extraction, and the remaining subsample was air‐dried for analysis of soil properties. The soil samples were kept at 4°C for no longer than 4 weeks before the incubation experiment began. Previous studies have shown that storing soil samples at 4°C for up to 7 weeks does not significantly affect microbial activity [49, 50].

### 3.2 Short‐Term Assay Experiment

Microbial CH_4_ oxidation rates in each of the forest soil samples (67 sites×4 replicates) were measured under sufficient substrate addition and optimal moisture conditions, employing previously developed methods for microbial thermal adaptation studies [23, 24]. For each site, 10 g of dry‐weight fresh soil was taken from each of the four replicates within sites and put into 140 mL incubation bottles. All subsamples were adjusted to 60% water‐holding capacity using sterile deionized water, which is optimal for microbial activity and gas diffusion. We preincubated these samples at 20°C under atmospheric CH_4_ concentrations for 3 days to restore methanotrophic activity from potential disturbances (e.g., soil sieving), as our preliminary experiments proved that this time frame was sufficient (Figure S3). Based on the growing season temperature range of the study site (Table S1), the soil incubations were performed at 15, 20 and 25°C because these incubation temperatures represent an appropriate temperature range for biotic activity of methanotrophs [7].

During the incubation, each bottle was sealed with a butyl rubber stopper, and pure CH_4_ was injected to approach a concentration of ∼200 ppm (Figure S4). This approach avoided the confounding effects of substrate limitation when assessing the thermal response of methanotrophic activity [23, 24]. Next, a 5 mL gas sample was collected using a syringe, followed by the immediate injection of 5 mL of CH_4_‐free air into the bottle to prevent fluctuations in air pressure within the incubation bottles. We kept 15°C bottles sealed for ∼3 h, 20°C bottles for ∼2 h, and 25°C bottles for ∼2 h. After the sealing period, we collected another 5 mL gas sample from the headspace and measured the CH_4_ concentrations by gas chromatography (Agilent 7890 A, Agilent Technologies Inc., Santa Clara, California, USA). Given the significant linear decline in headspace CH_4_ concentrations observed during these intervals (Figure S5), microbial CH_4_ oxidation rates were quantified based on the concentration change, soil dry weight, incubation duration, and headspace volume [6].

### 3.3 Thermal Incubation Experiment

Considering that methanotroph activity under atmospheric CH_4_ concentrations is generally substrate‐limited [8, 26], we used soils from 8 forest sites with different thermal regimes to conduct a thermal incubation experiment under different CH_4_ levels, aiming to investigate the effects of substrate availability on the thermal adaptation of microbial CH_4_ oxidation. The sites varied in climate, methanotroph communities, and soil properties (Table S1), allowing us to convincingly test how different CH_4_ levels influence thermal adaptation of microbial CH_4_ oxidation. In this study, two CH_4_ levels were established: 2 ppm (atmospheric) and 500 ppm (elevated). The incubation was carried out for 70 days, a duration expected to allow thermal adaptation of methanotrophs to occur, as soil microorganisms can undergo more than 100 generations during this period [10, 29], enabling genetic variation and shifts in community composition in response to the ambient thermal regime [10, 27, 29, 51].

For each site, 10 g dry‐weight soil from each of four replicates was placed in 140 mL bottles (8 sites×4 replicates×2 incubation temperatures×2 CH_4_ concentration treatments = 128 bottles). Soils were adjusted to 60% water‐holding capacity and pre‐incubated for 3 days at 20°C, then randomly assigned to 20 or 25°C. Soil water content was readjusted every 5 days throughout the incubation. For the atmospheric CH_4_ treatment, each incubation bottle was covered with a breathable membrane to allow continuous gas exchange with the surrounding atmosphere, ensuring that CH_4_ concentrations consistently remained at atmospheric levels throughout the 70 days. In contrast, for the 500 ppm CH_4_ treatment, soil samples were incubated in sealed bottles that were flushed with fresh air every 5 days to restore O_2_ levels and remove accumulated CO_2_. Immediately after each flushing, the headspace CH_4_ concentration was re‐adjusted to 500 ppm via pure CH_4_ injection. This periodic replenishment regime ensured that methanotrophic activity remained unaffected by substrate limitation throughout the 70‐day incubation, consistent with preliminary experiments showing that the initial 500 ppm CH_4_ supply was sufficient to sustain potential maximum oxidation rates for approximately 5 to 7 days across diverse soils (Figure S5).

At the end of the 70‐day incubation, we tested the thermal adaptation of soil microbial CH_4_ oxidation by conducting a short‐term assay experiment [14, 19]. Specifically, soils from each thermal treatment were split into three aliquots and placed at 15, 20, and 25°C. Microbial CH_4_ oxidation rates were measured within 24 h using the same procedure as in the initial assays. The brief duration of this measurement prevented microbial adaptation to the assay temperatures [14, 19]. Given the potential effects of generational turnover on methanotrophic biomass across different treatments, it was necessary to control for microbial biomass, as biomass is a key determinant of soil microbial metabolic rates [52]. Therefore, the thermal adaptation of soil microbial CH_4_ oxidation was evaluated on a mass‐specific basis [19, 23, 24]. To do so, CH_4_‐Oxi_mass_ was calculated by dividing the CH_4_ oxidation rates by the methanotrophic microbial biomass. We used the abundance of methanotrophic *pmoA* gene copies to estimate the biomass of methanotrophic microbes [6, 53, 54], as described below.

### 3.4 Climate Data and Soil Analysis

We extracted mean annual temperature and precipitation of 67 forest sites from the Worldclim dataset for the period 1970–2000 [55]. We measured soil total carbon (TC) using a TOC analyzer (Multi N/C 3100, Germany), soil total nitrogen (TN) using the Kjeldahl method [56]. Soil pH was measured at a 1:2.5 ratio of air‐dried soil to deionized water by a pH meter. Soil texture was analyzed using a particle size analyzer (BT‐9300ST, China).

### 3.5 Determining Microbial Communities and Biomass

Soil DNA was extracted from 300 mg of composite frozen samples from each site using the ALFA‐SEQ Magnetic Soil DNA Kit (Findrop Biosafety Technology (Guangzhou) Co. Ltd) following the instructions of manufacturer and was stored at ‐80°C for further applications. The *pmoA* gene is widely utilized for identifying methanotroph communities. In this study, the primers pmof1 (5′‐GGGGGAACTTCTGGGGITGGAC‐3′) and pmor (5′‐GGGGGRCIACGTCITTACCGAA‐3′) were used to amplify the methanotroph community from the eight long‐term thermal incubation sites. This *pmoA* primer has proven useful for amplifying both cultured and uncultured methanotrophs (Table S3). All PCR reactions were carried out in 50 µL volumes containing each primer (10 µM, Sangon) 1 µL, Taq DNA Polymerase 25 µL, and template DNA 50 ng. The thermal cycling protocol included an initial denaturation step at 95°C for 3 min, followed by 35 cycles of 95°C for 45 s, 55°C for 45 s, and 72°C for 45 s, with a final extension at 72°C for 8 min. The sequencing library was prepared and sequenced on an Illumina NovaSeq 6000 platform at Guangdong Magigene Biotechnology Co., Ltd, Guangzhou, China.

QIIME2 was used to process the raw sequences. DADA2, integrated within QIIME2, was employed to denoise the sequences into amplicon sequence variants (ASVs) after filtering out adaptor sequences, low‐quality reads, ambiguous nucleotides, and barcodes. Taxonomic assignment of the *pmoA* sequences was determined based on the FunGene database (Version 1.0) using the RDP Classifier with a confidence cut‐off of 80%. Rarefaction curves confirmed that the sequencing depth was sufficient to assess the diversity and community composition of the microbial populations in the soil (Figure S6). The sequence number in each sample was rarefied to the same depth for the *pmoA* gene (5,674) in subsequent comparative analyses.

We quantified the copy numbers of the *pmoA* gene using quantitative PCR (qPCR) with the primers pmof1/pmor. The final volume of the reaction system was 20 µL, including 10 µL SGExcel FastSYBR Mixture, 0.5 µL of each primer (10 µM, Sangon), 7.5 µL deionized H_2_O, and 1.5 µL template DNA. The thermocycler was programmed with an initial denaturation step at 95°C for 2 min, followed by 40 cycles of 95°C for 15 s, 55°C for 30 s, and 72°C for 30 s. The real‐time qPCR standards were obtained using a 10^4^–10^9^ gene copies µL^−1^ dilution series of plasmids containing *pmoA* gene fragments. Amplification efficiencies were 85%‐105% with R^2^ values higher than 0.99, and no signals were observed in negative controls. Based on the standard curves and the threshold cycle values, the *pmoA* gene copy numbers in the samples were calculated and reported per gram of dry soil. The abundance of *pmoA* gene copies was used to characterize the biomass of soil methanotrophs.

### 3.6 Calculating the Magnitude of Thermal Adaptation

To compare the capacities of microbial CH_4_ oxidation under atmospheric and elevated CH_4_ concentrations to adapt to long‐term temperature change, we calculated the magnitude of thermal adaptation (MTA) of microbial CH_4_ oxidation as follows:

(1) $$\mathrm{MTA}\;\left(\%\right)=\left(1-\frac{Oxi_{\mathrm{warmed}}}{Oxi_{\mathrm{control}}}\right)\times 100\;$$

where *Oxi*_warmed_ and *Oxi*_control_ represent microbial CH_4_ oxidation rates measured in soils incubated at 25 and 20°C, respectively. An MTA value <0 represents an absence of adaptation, whereas a value ≥0 indicates adaptation, with higher values corresponding to a higher degree of thermal adaptation.

### 3.7 Modelling the Relative Change in Soil CH_4_ Sink

To predict the effects of methanotroph thermal adaptation on soil CH_4_ sink across China's forests under a gradual increase of 2.0°C from 2023–2100, while accounting for differences in thermal adaptation under varying CH_4_ concentrations, we used two model scenarios—one for atmospheric CH_4_ concentration and the other for elevated CH_4_ concentration. The two scenarios were not designed as forecasts but as illustrations of how methanotroph thermal adaptation under diverse CH_4_ conditions could influence soil CH_4_ sinks under warming. We calculated changes in soil CH_4_ sinks for each year as follows:

(2) $$S_{j}=S_{j-1}\times \left(1-\Delta T\times A\right)$$

where *S*_*j*_ and *S*_*j*−1_ represent the soil CH_4_ sinks for the *j*th and (*j*‐1)th year, respectively. Δ*T* is the temperature increase for each year. *A* is the magnitude of thermal adaptation in atmospheric or elevated CH_4_ concentration treatments.

### 3.8 Statistical Analysis

Estimating shifts in microbial metabolic temperature responses along gradients of mean annual temperature is a classic ecological approach based on space‐for‐time substitution, reflecting biotic evolution and adaptation in response to temperature over long‐term timescales [57, 58]. To assess the effect of mean annual temperature on microbial CH_4_ oxidation rates, we established a linear mixed‐effects model (Table S2). Along with the thermal regime variables (mean annual temperature and assay temperature), we incorporated variables known to strongly influence microbial CH_4_ oxidation rates: soil total carbon, soil total nitrogen, clay content, and soil pH. To avoid the spatial autocorrelation of soil sampling sites, we considered the forest locations as a random effect. We used variance inflation factors (VIFs) to test for multicollinearity among the variables, but found VIFs < 2, indicating no evidence of multicollinearity (Table S4). Next, we estimated the relative effect size (slope) of each variable using a linear mixed‐effects model. Because thermal adaptation of the microbial community is defined as a decrease in microbial metabolic rates per unit biomass in response to a prolonged increase in temperature [14, 19], we used the mean methanotroph abundance across 67 sites to statistically control for methanotroph biomass in our regression model. This approach is equivalent to comparing microbial CH_4_ oxidation rate per unit biomass across all sites. Finally, based on the slope coefficients from the linear mixed‐effects model, the potential microbial CH_4_ oxidation rates along the broad thermal gradient were plotted against the site‐specific mean annual temperatures, with the microbial biomass and all other confounding variables held constant at their respective mean values. A linear mixed‐effects model was conducted using the “nlme” package [59].

We further used the linear mixed‐effects model to explore the impact of temperature increases on microbial CH_4_ oxidation rates at the assay temperature (20°C) under scenarios that assume both with thermal adaptation and without thermal adaptation, as numerous other studies investigating the influence of microbial thermal adaptation on soil carbon dynamics have done along a wide range of temperatures [23, 24]. To do so, we used the slope of the mean annual temperature coefficient from the linear mixed‐effects model (holding other factors constant) to represent the weakening of microbial CH_4_ oxidation rates caused by methanotroph thermal adaptation; we set this to zero in the scenario without thermal adaptation. These scenarios were not meant to predict future outcomes, as they could not capture the interactive effects of other variables; rather, their purpose was to elucidate microbial CH_4_ oxidation rates in response solely to temperature increases.

A two‐way ANOVA was used to assess the effects of incubation and assay temperature on microbial CH_4_ oxidation rates per unit biomass across different CH_4_ concentration treatments at each site. We performed paired sample *t*‐tests to compare the magnitude of thermal adaptation of microbial CH_4_ oxidation in atmospheric and elevated CH_4_ concentration treatments. Microbial diversity, including richness and Shannon index, was analyzed using the “vegan” package [60]. We evaluated the relative importance of mean annual temperature, mean annual precipitation, soil total carbon, soil total nitrogen, clay content, soil pH, and methanotroph alpha diversity (i.e., richness and Shannon index) in determining the magnitude of thermal adaptation of soil microbial CH_4_ oxidation using random forest analyzes with the “rfPermute” package [61]. All statistical analyses were performed using R statistical software (v.4.2.0).

## Author Contributions

H.C. developed the original idea; B.J. designed the research with assistance from H.C.; B.J. conducted the overall experiment and measurements with assistance from Z.Z., X.Z., and lab assistants; B.J. analyzed the data with assistance from H.C., N.H., and X.Z.; B.J. wrote the first draft, and H.C. and X.Z. revised the paper.

## Conflicts of Interest

The authors declare no conflicts of interest.

## Supporting information




**Supporting File**: advs77426‐sup‐0001‐SuppMat.docx.

## Data Availability

The data that support the findings of this study are openly available in Figshare at https://doi. orghttps://doi.org/10.6084/m9.figshare.30731045.
