# Supplementary material for: Constrained Forest Methane Sink by Thermal Adaptation of Soil Methanotrophs
Source: Adv Sci (Weinh). 2026 Aug 25:e77426. Online ahead of print. doi: 10.1002/advs.77426 (PMC13505580; doi:10.1002/advs.77426)
Supplement: Supplementary file 1 [file ADVS-9999-e77426-s001.docx]

Supporting Information for

**Constrained forest methane sink by thermal adaptation of soil methanotrophs**

**This file includes:**

Figs. S1 to S6

Tables S1 to S4.

**Supplementary Figures**

**Fig S1. Relationships between thermal adaptation magnitude of soil microbial CH_4_ oxidation and mean annual temperature (MAT) across 8 forest sites in atmospheric CH_4_ concentration (a) and elevated CH_4_ concentration (b).** Data are presented as mean values ± standard errors (n = 4).

**Fig S2. Effects of climate, soil microbial diversity, and soil properties on the magnitude of thermal adaptation of soil microbial CH_4_ oxidation.** **a, c** Results from random forest analysis displaying the relative importance of the explanatory factors in predicting the magnitude of thermal adaptation under the atmospheric CH_4_ concentration treatment (**a**) and the elevated CH_4_ concentration treatment (**c**). **b** Relationships between the magnitude of thermal adaptation and initial methanotroph richness in atmospheric CH_4_ concentration. **d** Relationships between the magnitude of thermal adaptation and soil total nitrogen content in elevated CH_4_ concentration. The shaded areas correspond to the 95% confidence intervals of the relationships. Data are presented as mean values ± standard errors (n = 4). MAT, mean annual temperature, MAP, mean annual precipitation, TC, soil total carbon content, TN, soil total nitrogen content, pH, soil pH, Clay, clay content, Initial_richness, initial methanotroph richness and Initial_shannon, initial methanotroph Shannon index.

**Fig S3. Temporal changes in microbial CH_4_ oxidation rates of forest soils incubated at 20°C with ample CH_4_ substrate and optimal moisture conditions.** Data are presented as mean values ± standard errors (n = 24).

**Fig S4. Response of soil microbial CH_4_ oxidation rates to increasing CH_4_ concentrations.** CH_4_ oxidation rates leveled off once the concentration exceeded 125 ppm, indicating that microbial methanotrophic activity was no longer limited by CH_4_ availability. Data are presented as mean values ± standard errors (n = 24).

**Fig S5. Temporal dynamics of headspace CH_4_ concentration during soil incubation at 20 °C under optimal moisture conditions.** The fitted solid line was estimated from a linear model, with the shaded region corresponding to the 95% confidence interval. Data are presented as mean values ± standard deviation (n = 24).

**Fig S6. Rarefaction curve of methanotroph community from the soil samples across different sites.** After rarefying the sequences to the same depth (5,674), the rarefaction curves approached a plateau for all sites, indicating that the sequencing depth was sufficient.

**Supplementary Tables**

**Table S1. Geographical information, climate, soil and microbial properties of the 67 forest sites.**

| Site name | LON (°) | LAT (°) | MAT  (℃) | MAP  (mm) | GST  (℃) | TC  (g/kg) | TN  (g/kg) | Clay  (%) | pH | *pmoA* gene copy numbers  (10^9^ copies g^-1^) | Initial methanotrophs  richness | Initial methanotrophs  Shannon index |
| --- | --- | --- | --- | --- | --- | --- | --- | --- | --- | --- | --- | --- |
| MHN | 122.52 | 52.7658 | -4.57 | 476 | 12.04 | 54.79±7.09 | 1.11±0.06 | 4.35±0.24 | 5.54±0.08 | 1.30 | / | / |
| ELCB | 122.94 | 51.1104 | -3.62 | 512 | 12.06 | 99.99±5.77 | 3.82±0.05 | 4.42±0.48 | 5.27±0.08 | 1.70 | / | / |
| MHB | 123.66 | 53.4486 | -3.57 | 469 | 13.24 | 49.09±2.90 | 0.98±0.30 | 5.16±0.33 | 4.85±0.04 | 5.62 | 51 | 4.41 |
| EWK | 120.45 | 47.7171 | -2.92 | 466 | 11.52 | 75.71±9.40 | 2.14±0.11 | 4.95±0.08 | 4.87±0.06 | 1.85 | / | / |
| ELCN | 122.38 | 49.6455 | -2.35 | 510 | 12.22 | 45.12±12.25 | 0.60±0.02 | 4.52±0.44 | 5.90±0.04 | 5.62 | / | / |
| YKS | 121.32 | 48.7485 | -2.04 | 463 | 10.58 | 46.66±1.09 | 0.88±0.04 | 5.21±0.41 | 5.71±0.06 | 5.50 | / | / |
| ECLD | 125.64 | 50.8355 | -1.88 | 515 | 12.18 | 54.20±4.39 | 1.26±0.03 | 5.03±0.10 | 6.21±0.20 | 18.25 | / | / |
| HM | 126.03 | 52.1452 | -1.63 | 496 | 12.63 | 44.05±3.05 | 0.67±0.02 | 9.16±0.30 | 5.93±0.03 | 1.97 | / | / |
| JGDQ | 124.47 | 50.2872 | -1.32 | 512 | 10.54 | 42.31±0.91 | 0.25±0.01 | 6.58±0.58 | 5.03±0.01 | 2.19 | / | / |
| HH | 128.64 | 48.3210 | 0.12 | 610 | 11.63 | 16.46±11.39 | 1.58±0.14 | 5.31±0.48 | 7.27±0.03 | 1.72 | / | / |
| ZSKT | 117.39 | 42.8592 | 0.47 | 422 | 10.04 | 39.53±5.91 | 2.18±0.17 | 1.77±0.43 | 5.86±0.05 | 2.60 | / | / |
| LX | 118.01 | 44.0846 | 0.99 | 381 | 10.83 | 87.00±6.85 | 1.46±0.05 | 3.06±0.53 | 6.21±0.10 | 1.58 | / | / |
| FYX | 133.77 | 47.8553 | 1.67 | 635 | 13.23 | 3.69±3.50 | 1.23±0.03 | 11.45±0.51 | 7.84±0.10 | 2.10 | / | / |
| FYD | 134.67 | 48.2799 | 1.91 | 692 | 13.17 | 32.87±0.65 | 0.42±0.06 | 2.90±0.27 | 6.96±0.14 | 1.09 | / | / |
| ZLTB | 119.95 | 45.2344 | 2.47 | 394 | 12.56 | 25.04±0.06 | 0.47±0.01 | 4.18±0.12 | 6.52±0.02 | 2.73 | / | / |
| FZ | 128.77 | 45.9243 | 2.73 | 566 | 14.03 | 21.83±0.68 | 0.58±0.02 | 4.03±0.23 | 6.49±0.08 | 2.02 | / | / |
| ZLQ | 122.22 | 47.1338 | 3.03 | 425 | 13.27 | 36.91±7.10 | 1.48±0.15 | 7.17±0.50 | 6.16±0.11 | 0.90 | 319 | 4.38 |
| WLCB | 112.65 | 40.8839 | 3.36 | 337 | 12.13 | 17.41±1.65 | 0.64±0.02 | 9.26±0.18 | 5.31±0.02 | 5.85 | / | / |
| ZLTD | 120.82 | 45.1339 | 3.40 | 405 | 12.97 | 41.99±3.49 | 1.42±0.03 | 6.63±0.97 | 7.45±0.09 | 1.26 | / | / |
| QQHE | 124.23 | 47.1937 | 3.54 | 424 | 14.57 | 25.30±1.35 | 1.02±0.09 | 4.93±0.20 | 6.83±0.10 | 2.82 | / | / |
| BQ | 132.13 | 46.7123 | 3.57 | 534 | 14.47 | 19.53±1.71 | 0.66±0.02 | 7.29±0.43 | 8.30±0.06 | 1.59 | / | / |
| TH | 126.09 | 42.0733 | 3.79 | 859 | 13.83 | 42.43±0.74 | 0.72±0.01 | 5.55±0.16 | 4.66±0.04 | 1.65 | / | / |
| JMS | 130.36 | 46.8234 | 3.82 | 599 | 14.69 | 21.66±1.58 | 1.89±0.06 | 5.58±0.91 | 6.28±0.57 | 1.79 | / | / |
| MS | 132.32 | 45.3649 | 4.02 | 573 | 14.26 | 7.97±1.49 | 1.89±0.04 | 6.59±0.12 | 5.13±0.33 | 3.11 | / | / |
| DQ | 125.17 | 46.5029 | 4.03 | 441 | 15.01 | 13.15±1.03 | 0.70±0.03 | 6.87±0.07 | 7.96±0.04 | 3.63 | / | / |
| HEB | 126.78 | 45.8839 | 4.07 | 521 | 15.13 | 5.49±1.08 | 1.12±0.12 | 6.30±0.27 | 5.75±0.04 | 4.30 | / | / |
| QGELS | 124.49 | 45.2242 | 4.89 | 455 | 15.73 | 61.51±5.11 | 0.95±0.10 | 1.15±0.69 | 4.68±0.57 | 1.86 | / | / |
| TY | 122.40 | 45.0668 | 5.97 | 413 | 16.16 | 13.63±2.06 | 0.83±0.03 | 8.03±0.43 | 6.10±0.11 | 2.66 | / | / |
| ZLTN | 120.59 | 44.4966 | 6.02 | 379 | 15.63 | 15.00±0.90 | 0.81±0.04 | 9.24±0.81 | 6.61±0.08 | 1.16 | / | / |
| BYZR | 108.74 | 40.7550 | 7.17 | 217 | 14.49 | 16.42±0.26 | 1.58±0.11 | 3.71±0.20 | 8.20±0.16 | 0.05 | / | / |
| SZ | 112.83 | 39.4725 | 7.44 | 393 | 14.00 | 19.59±2.43 | 0.71±0.10 | 10.22±0.16 | 4.57±0.20 | 1.16 | / | / |
| TL | 123.75 | 42.2783 | 7.61 | 632 | 15.06 | 22.29±6.91 | 0.41±0.10 | 4.42±0.65 | 7.33±0.08 | 5.93 | 524 | 7.31 |
| AK | 108.45 | 33.5866 | 8.17 | 806 | 11.54 | 16.61±0.88 | 0.35±0.01 | 4.29±0.30 | 7.77±0.29 | 0.94 | / | / |
| PJ | 121.91 | 40.9147 | 9.13 | 595 | 14.40 | 16.59±1.16 | 1.33±0.06 | 9.91±0.18 | 8.42±0.07 | 1.72 | / | / |
| DL | 121.45 | 39.6094 | 9.97 | 609 | 14.81 | 22.29±1.11 | 0.12±0.01 | 4.42±0.58 | 7.33±0.03 | 9.09 | / | / |
| QHD | 119.51 | 39.8507 | 10.50 | 595 | 15.28 | 61.51±36.70 | 0.21±0.11 | 1.15±0.46 | 4.68±0.06 | 1.33 | 193 | 4.91 |
| LY | 111.63 | 36.2490 | 12.62 | 493 | 15.46 | 71.09±0.60 | 0.89±0.03 | 5.09±0.32 | 5.33±0.02 | 0.76 | / | / |
| BD | 115.92 | 38.9136 | 12.92 | 489 | 15.98 | 18.73±0.38 | 3.37±0.21 | 17.73±0.43 | 8.24±0.02 | 10.68 | / | / |
| TJ | 117.48 | 38.7484 | 13.13 | 540 | 16.07 | 9.51±0.27 | 0.48±0.01 | 7.11±0.46 | 6.56±0.03 | 4.98 | / | / |
| XT | 114.42 | 36.9839 | 13.59 | 545 | 15.01 | 14.30±3.04 | 0.96±0.02 | 7.63±0.26 | 8.35±0.07 | 1.25 | / | / |
| LS | 119.61 | 27.7190 | 13.70 | 1858 | 13.70 | 10.26±0.51 | 0.06±0.01 | 4.67±1.16 | 8.29±0.13 | 1.23 | / | / |
| ZJ | 117.36 | 34.5993 | 14.04 | 679 | 15.35 | 15.21±2.21 | 0.71±0.05 | 4.19±1.39 | 7.54±0.07 | 0.60 | / | / |
| XX | 114.60 | 34.9482 | 14.19 | 612 | 15.49 | 3.51±0.69 | 1.84±0.15 | 6.35±0.32 | 7.03±0.02 | 2.43 | / | / |
| SZ | 118.83 | 33.2928 | 14.53 | 852 | 14.53 | 13.61±2.43 | 0.34±0.02 | 4.59±0.16 | 7.85±0.20 | 1.14 | / | / |
| ZK | 114.90 | 33.4809 | 15.03 | 799 | 15.03 | 13.45±0.91 | 0.27±0.01 | 12.08±1.32 | 5.44±0.07 | 1.36 | / | / |
| ZY | 106.88 | 27.7626 | 15.30 | 1060 | 15.30 | 6.85±0.99 | 0.29±0.03 | 10.87±0.38 | 8.07±0.11 | 2.11 | / | / |
| NY | 112.65 | 33.2622 | 15.33 | 840 | 15.33 | 18.49±5.33 | 1.68±0.23 | 2.40±0.37 | 8.43±0.09 | 1.60 | 228 | 4.41 |
| FY | 116.29 | 32.5713 | 15.47 | 918 | 15.47 | 14.13±1.69 | 0.32±0.04 | 2.83±0.39 | 5.35±0.11 | 0.94 | / | / |
| YX | 102.95 | 24.5996 | 15.52 | 888 | 15.52 | 18.46±1.47 | 1.25±0.20 | 12.27±0.21 | 6.07±0.42 | 1.57 | / | / |
| WSZZ | 104.30 | 23.5752 | 15.80 | 1310 | 15.80 | 18.08±2.31 | 0.16±0.04 | 3.07±0.78 | 3.23±0.02 | 0.75 | / | / |
| YC | 112.06 | 30.9595 | 16.16 | 1011 | 16.16 | 7.94±6.09 | 0.87±0.01 | 11.50±5.39 | 7.33±0.09 | 0.76 | / | / |
| SY | 111.49 | 32.5260 | 16.23 | 944 | 16.23 | 13.19±5.81 | 1.01±0.26 | 13.59±0.55 | 7.53±0.01 | 1.13 | / | / |
| CZ | 117.05 | 30.4104 | 16.73 | 1505 | 16.73 | 30.10±4.89 | 1.11±0.09 | 11.76±3.16 | 8.34±0.06 | 0.59 | / | / |
| HZ | 119.06 | 29.6074 | 16.80 | 1495 | 16.80 | 18.53±1.20 | 0.12±0.11 | 11.84±0.41 | 7.99±0.08 | 1.61 | / | / |
| EZ | 114.64 | 30.2361 | 17.02 | 1320 | 17.02 | 15.96±0.75 | 0.56±0.05 | 12.29±0.35 | 7.23±0.21 | 0.78 | / | / |
| ZJJ | 110.56 | 29.1298 | 17.17 | 1365 | 17.17 | 6.85±3.55 | 0.66±0.48 | 10.87±2.14 | 8.07±0.05 | 1.48 | 248 | 5.53 |
| JDZ | 116.33 | 28.8954 | 17.56 | 1563 | 17.56 | 19.00±2.40 | 1.46±0.20 | 0.66±0.91 | 8.17±0.46 | 0.63 | / | / |
| CQ | 106.67 | 29.0221 | 17.94 | 1116 | 17.94 | 5.75±1.73 | 1.27±0.18 | 10.33±0.13 | 7.68±0.57 | 0.78 | / | / |
| GZ | 115.03 | 25.8832 | 19.33 | 1454 | 19.33 | 11.66±3.11 | 0.36±0.06 | 7.10±5.52 | 5.96±0.53 | 5.49 | / | / |
| GL | 110.21 | 25.0913 | 19.53 | 1841 | 19.53 | 27.17±3.02 | 1.90±0.14 | 10.76±1.09 | 5.53±0.53 | 1.31 | / | / |
| MH | 100.26 | 22.0316 | 20.19 | 1548 | 20.19 | 2.17±0.80 | 1.39±0.16 | 6.83±0.07 | 7.45±0.43 | 1.73 | / | / |
| LD | 108.86 | 18.7407 | 20.77 | 1509 | 20.77 | 18.70±0.88 | 1.17±0.08 | 4.52±0.05 | 7.26±0.57 | 3.11 | / | / |
| LB | 109.18 | 23.7673 | 21.24 | 1500 | 21.24 | 7.94±0.84 | 0.05±0.01 | 11.50±1.01 | 7.33±0.90 | 1.09 | 275 | 6.11 |
| BS | 107.59 | 23.2997 | 22.43 | 1551 | 22.43 | 10.28±0.88 | 0.18±0.04 | 11.64±0.55 | 6.42±0.05 | 0.47 | / | / |
| ML | 101.27 | 21.9255 | 22.65 | 1649 | 22.65 | 19.82±0.18 | 0.13±0.02 | 7.30±0.61 | 3.66±0.17 | 0.66 | / | / |
| JH | 100.83 | 21.9874 | 23.11 | 1652 | 23.11 | 32.05±0.83 | 0.09±0.01 | 9.43±1.24 | 4.46±0.11 | 0.17 | / | / |
| TC | 110.11 | 19.4041 | 24.19 | 1713 | 24.19 | 10.71±1.70 | 0.27±0.02 | 9.09±1.02 | 5.47±0.43 | 0.27 | 196 | 5.30 |

LAT, latitude; LON, longitude; MAT, mean annual temperature; MAP, mean annual precipitation; GST, growing season temperature; TC, soil total carbon content; TN, soil total nitrogen content; pH, soil pH; Clay, clay content. Soil samples from 8 different ambient thermal regime sites (MHB, ZLQ, TL, QHD, NY, ZJJ, LB, TC) were incubated at 20 and 25 °C for 70 days under two CH_4_ concentration treatments. Data are presented as mean values ± standard errors (n = 4).

**Table S2. Coefficients (mean ± SD) and R^2^ values for the linear mixed-effects model used to evaluate the influence of long-term temperature change on soil microbial CH_4_ rates.**

| Variables | Coefficient |
| --- | --- |
| Intercept | 1.2027±1.4076 |
| Assay temperature | **0.1542±0.0131** |
| MAT | **-0.0716±0.0342** |
| *pmoA* gene copy number | 0.0444±0.0811 |
| TC | **0.0390±0.0130** |
| TN | 0.0753±0.3264 |
| pH | -0.1635±0.1694 |
| Clay | 0.0127±0.0632 |
| Marginal R^2^ | 0.39 |
| Conditional R^2^ | 0.90 |

MAT, mean annual temperature; TC, soil total carbon content; TN, soil total nitrogen content; pH, soil pH; Clay, clay content. Significant (*P* < 0.05) coefficients are shown in bold.

**Table S3. The taxonomic information of aerobic methanotrophs identified in this study.**

| Taxonomy of aerobic methanotrophs | Genus |
| --- | --- |
| Upland soil cluster Alphaproteobacteria  (Type II) | *Methylocystis*  *Methylosinus*  *Methylocapsa*  *Methylocella* |
| Upland soil cluster Gammaproteobacteria  (Type Ⅰ) | *Methylobacter*  *Methylomicrobium*  *Methylohalobius*  *Methylosoma*  *Methylothermus*  *Clonothrix*  *Methylomonas*  *Methylosarcina*  *Methylovulum*  *Methylomarinum*  *Crenothrix*  *Methylocaldum*  *Methylococcus*  *Methylogaea* |

**Table S4. Correlation of fixed effects and the square-root of the predictor variable inflation factors (VIFs)** **for the linear mixed-effects model** **reported in Table S2.**

|  | Variables | | | | | | | VIFs |
| --- | --- | --- | --- | --- | --- | --- | --- | --- |
|  | Assay temperature | MAT | *pmoA* gene copy number | TC | TN | pH | Clay |  |
| Assay temperature | NA | NA | NA | NA | NA | NA | NA | 1.000 |
| MAT | 0.000 | NA | NA | NA | NA | NA | NA | 1.662 |
| *pmoA* gene copy number | 0.000 | 0.286 | NA | NA | NA | NA | NA | 1.131 |
| TC | 0.000 | 0.383 | 0.077 | NA | NA | NA | NA | 1.597 |
| TN | 0.000 | 0.189 | -0.099 | -0.240 | NA | NA | NA | 1.227 |
| pH | 0.000 | -0.054 | -0.002 | 0.294 | -0.126 | NA | NA | 1.153 |
| Clay | 0.000 | -0.274 | -0.082 | 0.128 | -0.205 | -0.001 | NA | 1.185 |

MAT, mean annual temperature; TC, soil total carbon content; TN, soil total nitrogen content; pH, soil pH; Clay, clay content.
